# ‘Water is life’: developing community participation for clean water in rural South Africa

**DOI:** 10.1136/bmjgh-2018-001377

**Published:** 2019-06-11

**Authors:** Jennifer Hove, Lucia D'Ambruoso, Denny Mabetha, Maria van der Merwe, Peter Byass, Kathleen Kahn, Sonto Khosa, Sophie Witter, Rhian Twine

**Affiliations:** 1 MRC/Wits Rural Public Health and Health Transitions Research Unit (Agincourt), School of Public Health, Faculty of Health Sciences, University of the Witwatersrand, Johannesburg, South Africa; 2 Institute of Applied Health Sciences, School of Medicine, Medical Sciences and Nutrition, University of Aberdeen, Aberdeen, Scotland, UK; 3 Centre for Global Development, School of Education, University of Aberdeen, Aberdeen, Scotland, UK; 4 Department of Epidemiology and Global Health, Umeå University, Umeå, Sweden; 5 Department of Health, Mpumalanga Provincial Government, Mbombela, South Africa; 6 Institute for Global Health and Development, Queen Margaret University Edinburgh, Musselburgh, Scotland, UK

**Keywords:** water, south africa, participatory action research, photovoice, rural

## Abstract

**Background:**

South Africa is a semiarid country where 5 million people, mainly in rural areas, lack access to water. Despite legislative and policy commitments to the right to water, cooperative governance and public participation, many authorities lack the means to engage with and respond to community needs. The objectives were to develop local knowledge on health priorities in a rural province as part of a programme developing community evidence for policy and planning.

**Methods:**

We engaged 24 participants across three villages in the Agincourt Health and Socio-Demographic Surveillance System and codesigned the study. This paper reports on lack of clean, safe water, which was nominated in one village (n=8 participants) and in which women of reproductive age were nominated as a group whose voices are excluded from attention to the issue. On this basis, additional participants were recruited (n=8). We then held a series of consensus-building workshops to develop accounts of the problem and actions to address it using Photovoice to document lived realities. Thematic analysis of narrative and visual data was performed.

**Results:**

Repeated and prolonged periods when piped water is unavailable were reported, as was unreliable infrastructure, inadequate service delivery, empty reservoirs and poor supply exacerbated by droughts. Interconnected social, behavioural and health impacts were documented combined with lack of understanding, cooperation and trust between communities and authorities. There was unanimity among participants for taps in houses as an overarching goal and strategies to build an evidence base for planning and advocacy were developed.

**Conclusion:**

In this setting, there is willingness among community stakeholders to improve water security and there are existing community assemblies to support this. Health and Socio-Demographic Surveillance Systems provide important opportunities to routinely connect communities to resource management and service delivery. Developing learning platforms with government and non-government organisations may offer a means to enable more effective public participation in decentralised water governance.

**Key questions**

**What is already known?**

- While the South African government recognises water as a basic human right and mandates participatory water governance, authorities lack the means to authentically engage communities and the capacities to use community-generated evidence in resource allocation.

**What are the new findings?**

- Continuous water shortages are an overwhelming impediment driving people into poverty, illness and social unrest. Lack of understanding between communities and authorities entrenches the situation.
- The process was an acceptable means of capturing the multidimensional nature of the problem as a first step towards using this information in planning and advocacy.

**What do the new findings imply?**

- Routine community-led accountability and awareness raising engaged with authority bodies may help to develop interfaces, trust, more effective monitoring and improved service provision.
- Demographic surveillance provides opportunities to develop fuller, routine forms of participatory deliberation and decision making.

## Background

In rural communities around the world, lack of clean water and sanitation are major contributors to avoidable death and disease. Lack of clean water increases vulnerability to conditions including diarrhoea, malnutrition, malaria, lymphatic filariasis, intestinal nematode infections, trachoma and schistosomiasis.^1^ In 2012, 742 000 diarrhoea-related deaths were caused by lack of access to water and sanitation.^2^ The impact of diarrhoea is most acute in children under 5 years and is a leading cause of death in this age group.^3^

The importance of water was emphasised in the Millennium Development Goals during which time access expanded considerably. The target for safe drinking water was achieved in 2010 ahead of the 2015 deadline with 91% of the world population accessing safe drinking water, compared with 76% in 1990.^4^ An estimated 663 million people still lacked access in 2015, however, and the target on improved sanitation was not met.^5^ The Sustainable Development Goals 2015–2030 commit to the unfinished water agenda, aiming to ensure available and sustainable management of water and sanitation for all.^6^

In postapartheid South Africa, the government enshrined water as a basic human right in the 1996 constitution, 14 years before the 2010 UN declaration.^7 8^ The National Water Act of 1998 and the Water Services Act of 1997 were among a series of legislative and policy shifts to redress discrimination, promote equitable access and support municipalities to provide services, in which cooperative governance and public participation were centralised.^9–11^ Between 1994 and 2004, the government invested 15 billion ZAR (US$3 billion) in infrastrucutre.^12^ From 1990 to 2015, access increased 98%–100% and 66%–81% in urban and rural areas, respectively.^13^

While initially driven centrally, in 2003 and 2006, service provision and water supply were devolved to local governments and municipalities, respectively.^12 14^ Ambitious decentralisation and developmental agendas relocated a range of responsibilities to local levels, where multiple constraints were faced. These included: financial distress, debt, inability to raise revenue, serious administrative and financial mismanagement, neo-patrimonialism, tendering corruption and manipulation of public procurement.^15 16^ Municipalities were also unable to account for large amounts of complex technical information, with no monitoring systems for household usage in most rural areas.^17^

Community-based water management was assigned to Catchment Management Agencies (CMAs) promoting public participation and Water User Associations (WUAs) as user cooperatives.^18^ There were challenges with capacity and clarity, however, with overlapping and ill-defined mandates, and links to community-based structures were limited.^19^ While CMAs and WUAs were ‘*meant to increase participation of stakeholders including communities in the management of water resources… efforts have not translated into effective participation… there is no link between the national water quality management frameworks and community-based development structures*’.^20^ Of the 19 CMAs established nationally, only two were operational in 2015.^21^

As well as advancing nation-building, the government embraced the international Integrated Water Resource Management (IWRM) paradigm.^21^ Over time, IWRM came to be seen as overly complex and technocratic, however, limiting the state’s role, with insufficient attention to context, integration and overlooking poverty alleviation.^22^ This was followed by calls for a renewed focus on context in policy, strategic intervention and resource mobilisation with community participation as a unifying concept.^23 24^ The second National Water Resource Strategy acknowledges the role of water in social and economic development and commits to infrastructure, services and equity as a policy goals.^25^

Despite visionary policy, legislation and investment in infrastructure, maintenance backlogs have become a systemic challenge. In 2012, costs for outstanding maintenance reached US$1.4 billion, and there are high proportions of households (78% in Mpumalanga and 70% in Limpopo) without basic services and interrupted supply due to non-functioning, poorly maintained infrastructure and empty or insufficiently supplied reservoirs.^12 26^ Today, around 5 million South Africans, mainly in rural areas, do not have reliable access to drinking water.^27^

The human and societal costs are extensive. In addition to avoidable mortality, studies have identified risks of physical disability due to water carrying, a burden borne disproportionately by women and children.^28 29^ Shame and emotional distress have been related to lack of water as has the erosion of social cohesion and capacity for community participation.^30 31^ Service delivery protests have also increased drastically, associated with violent masculinities and a crisis of representation in local government.^32–34^

Despite normative support for participatory water governance, authorities lack the means to effectively consult communities and the capacities to use community-generated data and evidence in water resource management and service delivery. Innovations in community interfaces with health and water authorities are therefore urgently required.

### Aims and objectives

The aims were to elicit local knowledge on health priorities in a rural province in South Africa as part of a wider process developing community evidence for policy and planning. This paper reports on the first element, developing community-generated evidence for action. The objectives were to elicit local insights into community-nominated priorities and develop actions to address the issues identified.

## Methods

**Figure 1:**
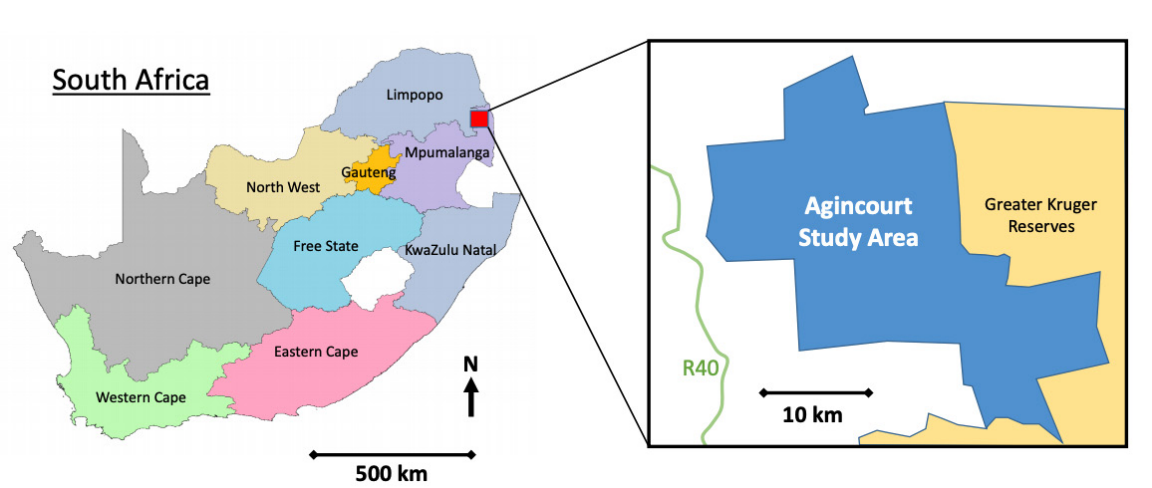
Map of Agincourt Health and Socio-Demographic Surveillance System research area

The research was based at the Agincourt Health and Socio-Demographic Surveillance System (HDSS) in Mpumalanga, northeast South Africa (figure 1). The HDSS is one of Africa’s largest population-based cohorts conducting annual updates on vital events including births, deaths, migration and socioeconomic status and has led efforts to develop HDSS infrastructure nationally. The area is densely populated with 116 000 people in 18 500 households in 31 villages covering 420 square kilometres and 30% of the population comprises former Mozambican refugees.^35^ There is little formal sanitation, and electricity is available but affordable to a minority.^36^ There is high unemployment and a limited economic base resulting in labour migration and significant reliance on social grants. While socioeconomic status has improved 2001–2013, it has been slower for poor households.^37^

We adopted a participatory action research (PAR) process with community stakeholders in the Agincourt HDSS area. PAR is an approach focused on inclusion and social action through mutual transfer of expertise, power and ownership towards those most directly affected by the issues under investigation. PAR has a cyclical nature with repeated rounds of collective analysis, taking and evaluating action and learning from action.^38^ This paper reports on PAR with community stakeholders as part of a wider programme developing community evidence for policy and planning (www.vapar.org). To our knowledge, this work is the first to develop participatory decision making to improve access to clean, safe water in a demographic surveillance platform.

We began by attempting to re-engage 22 participants involved in earlier participatory research across three villages in the Agincourt HDSS.^39–41^ (The term ‘participants’ refers to people engaged in the PAR process as coresearchers rather than passive research subjects. We use the term synonymously with ‘community stakeholders’.) In the earlier work, villages were selected to vary by distance to health facilities and levels of child-headed households. In each village, participants had been selected to represent service users and providers. Individuals were contacted by telephone and/or approached in their localities. From the 22 original participants, 13 agreed to be involved (nine were unable due to moving away, work commitments and one had passed away). Where it was not possible to find or re-engage those involved in the earlier work, 11 individuals with similar characteristics were approached and recruited using the same approach.

In each village, we held an initial workshop where we introduced the present study. We invited participants to codesign the process by nominating the health issues to examine and by expanding the participant base. Lack of water was a priority issue identified in one group (n=8 participants) and alcohol and other drug (AOD) abuse was nominated in the other two (n=16 participants). This paper focuses on water, the findings on AOD abuse are presented elsewhere.^42^ Women of reproductive age (WRA) were nominated as a group affected by, and with important knowledge on, lack of water, and new participants (n=8) were nominated by participants and recruited by the research team (new participants were also recruited in the two groups nominating AOD abuse). Table 1 provide details of the original and new participants in the group nominating water and for the three groups overall.

**Table 1:** Composition of discussion groups

Village ‘A’ nominating water (original and new participants denoted as A1 and A2, respectively)			
Selection criteria/role in community	Original participants (A1)	New participants (A2)	Total
Traditional healers	1		1
Community health volunteers	1		1
Community officials	2		2
Family members	3	3	6
Women of reproductive age	1	5	6
Total number of participants	8	8	16
Proportion female (%)	63	100	81

Villages ‘A’, ‘B’ and ‘C’ (original and new participants denoted as A1, B1, C1 and A2, B2 and C2, respectively)			
Selection criteria/role in community	Original participants (A1, B1 and C1)	New participants (A2, B2 and C2)	Total
Traditional healers	3		3
Religious leaders	1		1
Community health volunteers	3		3
Community officials	5		5
Family members	7	3	10
Women of reproductive age	5	5	10
Youth		16	16
Total number of participants	24	24	48
Proportion female (%)	75	92	83

All participants were 18 years or older. Participants were acknowledged as having multiple roles at home and in the community and a primary role was identified with participants for the purposes of recruitment.

Following the introductory workshop (workshop 1), we held a series of workshops that were structured and sequenced to systematise subjective perspectives into shared forms of knowledge on the nature of the problem and build consensus on actions to address the issues identified. The sequence progressed as follows:

- In workshop 2, participants deliberated over water shortages using a ‘problem tree’ to identify cause-and-effect relationships at various levels, building shared accounts, identifying and relating relevant social, behavioural and health factors.
- In workshop 3, using the problem tree, ‘Venn diagrams’ were developed to build shared understandings of the relationships between key actors and institutions.
- In workshop 4 with reference to the problem tree and Venn diagrams, ‘action plans’ were developed using stepwise pathways specifying actors, actions and outputs to achieve agreed goals.

Following workshops 1–4, we held additional workshops in which participants across all villages came together to revisit and cross-validate each other’s findings and to reflect on the experience and how the process could be carried forward with government and non-governmental organisations (NGOs). In these workshops, participants were encouraged to adopt roles as cofacilitators in the deliberations. We employed a visual method (Photovoice) as a further input to the discussions (table 2).^43^ Participants were provided with digital cameras to record lived realities of water insecurity. In workshops 3–8, participants presented and discussed images and developed captions to describe what the images conveyed. In each workshop, there was also discussion and reflection on PAR principles of representation, lived experience, ownership and collective knowledge (table 3).

**Table 2:** Schedule of community stakeholder workshops for village A*

Work-shop	Villages	Weekly meeting topics	Tool/technique	Description
1	A1	Topic selection	Ranking and voting	To identify priority health topic of relevance to the community. A list of health priorities was developed during the discussion, after which participants voted for the topics of highest relevance using adhesive stickers. The voting progressed through two rounds with discussion and agreement at the end.
2	A1, A2	Problems and causes	Problem tree	To unpack/understand nominated topics from different perspectives. Through facilitated discussions using a tree diagram visible to all, participants identified cause-and-effect relationships at various levels from root (tree roots) to intermediary causes (trunk and branches) and consequences and other effects (tree pods) building subjective perspectives into shared accounts through consensus.
3	A1, A2	Actors and impacts	Venn diagrams	To understand impacts and actors. Collective account developed with Venn diagram made from cardboard circles of different sizes and colours to indicate relationships and interactions between various actors and institutions, identifying internal and external organisations active in the topic and how they related to one another in terms of contact and collaboration.
4	A1, A2	Action agendas	Action pathways	To articulate overall goal(s) to address issues identified and visualise and depict stepwise actions and actors to achieve these. The action pathway was collectively developed to represent moving towards a desired goal via a series of interconnected events.
5	ABC	Problems and causes	Problem tree	As per workshop 2.
6	ABC	Actors and impacts	Venn diagrams	As per workshop 3.
7	ABC	Action agendas	Action pathways	As per workshop 4.
8	ABC	Reflections and next steps	Facilitated discussion	To reflect on experiences, outputs and how the process should be carried forward to engage government and non-governmental organisations. Participants discussed differences and similarities between the workshop outputs through facilitated discussions, cross-verified each other’s outputs and reflected on the process and future development.
1–8	ABC	Lived experience	Photovoice	To visually convey lived expereince. Participants given basic training in photography, research ethics and digital cameras to take photographs illustrating the topic or condition as it existed in the physical environment. Photographs presented and discussed in meetings and captions developed to describe what images conveyed.

A1: original participants village ‘A’; A2: new participants village ‘A’; ABC, three villages combined.

*The table presents the process for village ‘A’, nominating water. All village-based discussion groups ‘A’, ‘B’ and ‘C’ progressed through this sequence independently, coming together for workshops 5–8 to further build consensus, verify outputs and reflect on process and next steps.

**Table 3:** PAR principles reinforced in each community stakeholder workshop

Principle	Description
No delegation	Participants are those directly affected and are the primary researchers taking lead roles forming teams to identify problems, define, analyse and develop solutions.
Homogeneous group	A social group with shared conditions to discuss, deliberate and reach consensus on the nature of the problem and actions to address the issues identified.
Subjective perspectives	People’s individual experiences are central to the process and are the foundation on which collective knowledge is developed, respecting each other’s opinion, as opposed to imposing ideas/opinions on others.
Collective validation	Recording observations that all participants identify as important. Does not negate differences in perceptions and experiences, but encourages the group to reach consensus on collective findings through corroboration of information and experiences.

Loewenson *et al* 2014.^38^

PAR, participatory action research.

Workshops were held in the common local language xiTsonga, with some content in English. Topic guides in xiTsonga were used to structure discussions, which were facilitated by a researcher familiar with the area (DM). Discussions were recorded on prepared flip charts to display a collective record (DM, JH, RT and LD), allowing for checking and rechecking of consensus views. Researchers cofacilitated the meetings, provided general assistance and took observational notes (JH, RT and LD). With separate permissions, workshops were audio-recorded, transcribed verbatim and translated into English. A 10% sample was back-translated for quality assurance. Data were stored in audio recordings, Microsoft Word and image files. Data were managed by researchers and stored on secure University servers.

Analysis was performed during the workshops by participants. Findings were collectively validated, with outputs recorded and appraised. During and after data collection, thematic analysis of narrative and visual data was performed to document and disseminate the community stakeholders’ analyses. One researcher (JH) reviewed the transcripts in detail to identify recurring patterns with regular checking and cross-checking (LD and RT). A combined inductive/deductive approach was adopted.^44^ Deductive categories were drawn from the process of understanding and articulating the problem to developing solutions and inductive themes were generally, although not exclusively, arranged according to this sequence. Thematic codes were developed and organised until no new themes emerged. Visual data were also reviewed and assigned codes (JH, DM and LD).^45^ The following section presents the results of the analysis.

Specific ethical considerations applied to the PAR. PAR is founded on a position in which realities are subjective, contested, multiple and social and where knowledge is concerned with transferring power towards those most directly affected. Ethical conduct is therefore considered in terms of power dynamics by those intended to benefit from the process. During data collection and analysis, there was regular attention to power dynamics with time spent in each workshop, and in a dedicated workshop at the end of sequence, reflecting on these categories. Participants using visual methods received basic training in photography and on how to secure release permissions from the subjects of images.

Otherwise participants were informed about the nature of the research, its aims, procedures and outcomes before agreeing to be involved and were assured anonymity in the reporting of identifiable information with an option to opt-out. Participants were reimbursed for time spent in the process via subsistence and travel expenses (200 ZAR, US$13 per participant). Refreshments were also provided in the workshops. Participants were free to leave the study at any time and for any reason. Preliminary results were fed back to, and verified with, participants before being disseminated more widely.

### Patient and public involvement

The research was developed to address health priorities identified by community stakeholders. We did not work with patients directly but with people living in rural villages who were involved in study design in terms of nominating focus topics and expanding the participant base. Community stakeholders were integral to the process of systematising subjective perspectives into collective forms of knowledge as a basis to leverage action and learning from action. The results of the study will be used as the basis of subsequent engagements with government organisations and NGOs, and in which community stakeholders will be centrally involved.

## Results

**Figure 2:**
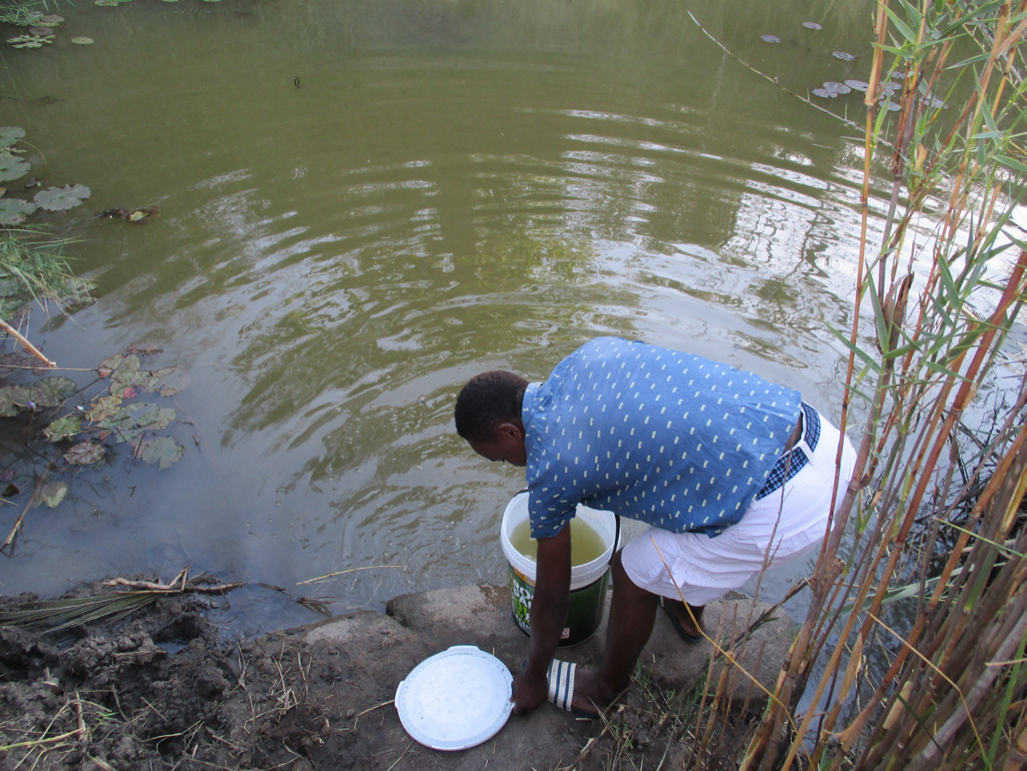
A man fetches water at the river for household use. Community stakeholder description: this picture illustrates the circumstances that people face every day. Despite the risks of using dirty water, people are left with no option but to use dirty water to which they have access.

**Figure 3:**
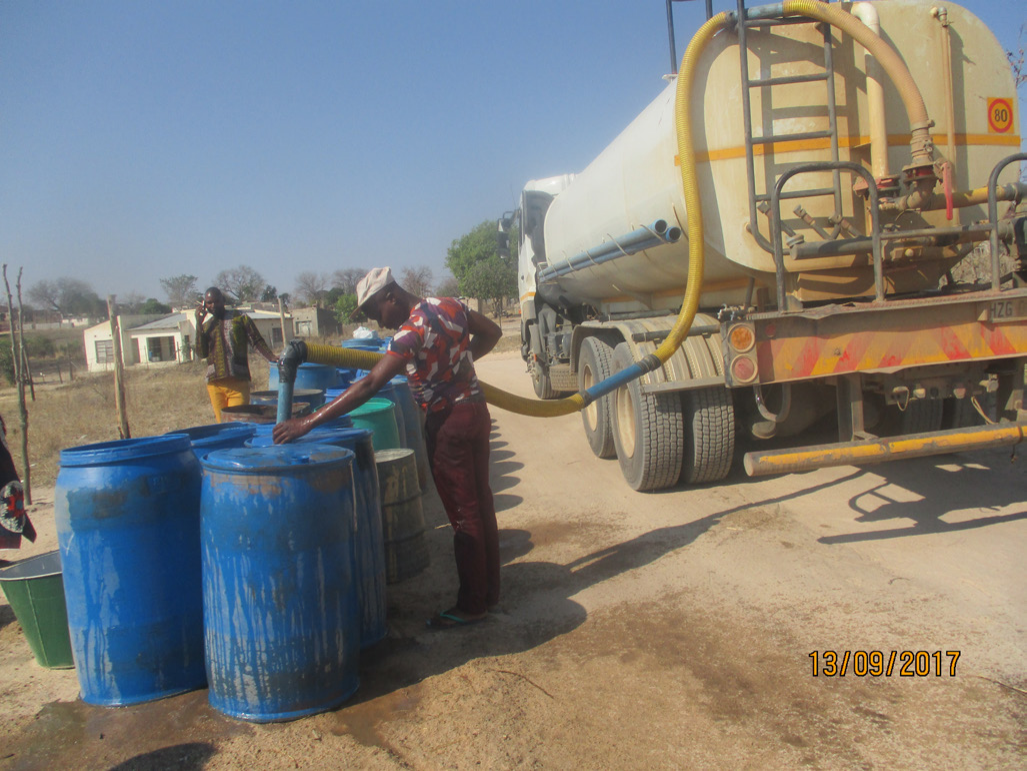
A tanker that supplies villages with water due to intermittent supply. Community stakeholder description: the community see this as an impediment to provision of domestic taps, deterring attention from efforts to improve the water situation. Furthermore, mobile tankers are not always available due to lack of fuel and are alleged to deliver contaminated water.

**Figure 4:**
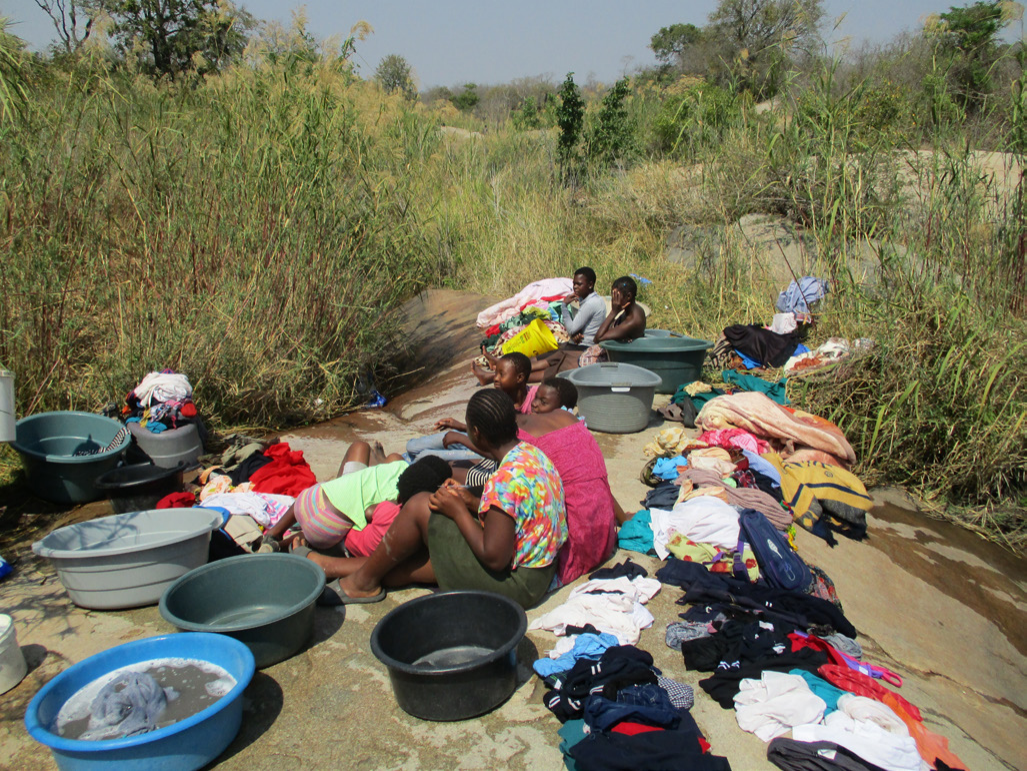
Women accompanied by children doing laundry by the river. Community stakeholder description: laundry is done on ‘washing rocks’ or in dishes because there is no running water in the communal taps. Doing laundry by the river saves carrying water to households.

**Figure 5:**
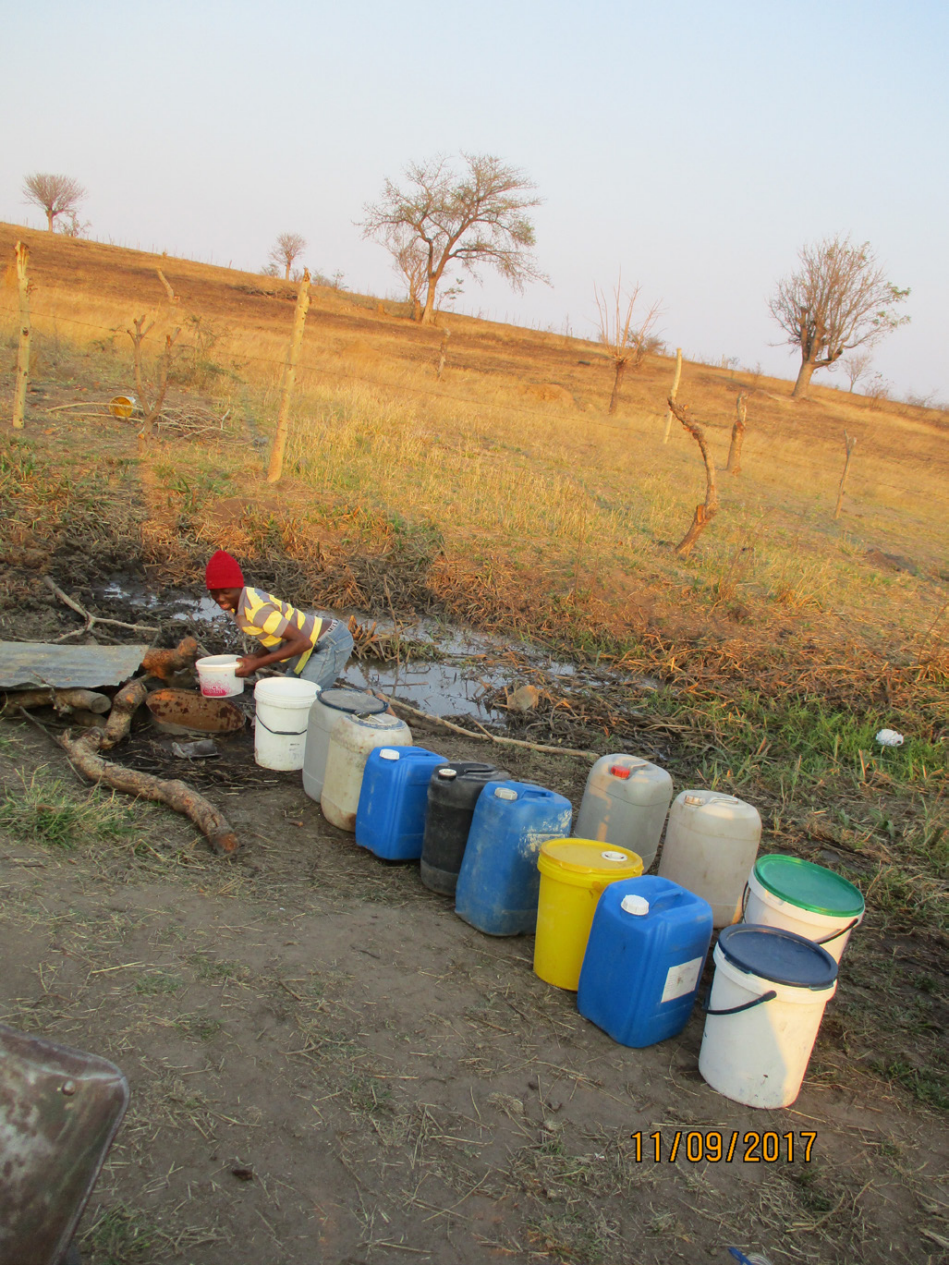
Female community member fetching water alongside a string of water containers. Community stakeholder description: the containers are left in the queue to be filled when the water returns. People spend large amounts of time waiting for water at unreliable sources.

The analysis of narrative and visual data is presented below, and in table 4 and figures 2–5.

**Table 4:** Thematic framework

Theme	Subtheme
Problem definition: lack of household supply, alternative and unregulated sources.	Repeated/prolonged periods without domestic supply. Informal/unregulated sources – water tankers provided by municipality. Informal/unregulated sources – cement/traditional wells/boreholes/surface water. Inconvenience of collection from informal/unregulated sources. Domestic storage of water from informal/unregulated sources, contamination and damaging equipment. Lack of political attention to the problem.
Causes and contributors of water shortages	Poor governance and planning and lack of awareness in authorities. Lack of political accountability (‘broken election promises’) and corruption. Lack of awareness of accountability mechanisms among community leaders (CPF, CDF, Induna, councillors and ward committees). Lack of infrastructure maintenance and delays in maintenance. Vandalism and limited community ownership. Persistent droughts, high temperatures and low rainfall.
Health and social impacts	Avoidable infectious disease and mortality, waterborne diseases: schistosomiasis, cholera, typhoid and other intestinal infectious conditions. Sanitation compromised without clean water. Hunger and malnutrition: diminished possibilities to grow/prepare food. Economic impacts: time costs to access water and necessary to buy water from tankers. Safety concerns: women collecting water at night and early morning. Familial/educational impacts: parents/children walking long distances to collect water. Personal and social impacts: continuous struggle, personal unhappiness and stress, neighbourhood fights, hatred and division and violent community protests.
Priorities for action	Ensure household provision of water via taps in households (overall goal). Improve reporting systems on extent of problem for planning and advocacy: Inventories of households without water. Detailed monitoring of water-related challenges in the community. Fund-raising for monitoring, planning and infrastructure development. Fairer allocation of resources, multisectoral deliberation and partnerships. Strengthen relationships between community structures (community leaders and ward committees) and water management and service delivery authorities. Enable community participation with local government/municipalities in water supply. Strengthen infrastructure and maintenance and advance technologies. Encourage collective responsibility in communities: protection of catchment areas and protect water from contamination. Community awareness campaigns and education.
Reflections on the process	Collective experience to understand complex topic. Shared benefit and exchange of understanding. Benefit of principle of respect and valuing participants. Expectations raised for future action. Dissatisfaction over level of reimbursement.

Community Police Forum (CPF): a group from communities representing police who meet to discuss safety in communities. They aim to ensure police accountability, transparency and effectiveness. CPFs are established in terms of section 19(1) of the SAPS Act, Act 68 of 1995 (Source: RSA. No. 68 of 95 South African Police Service Act. Pretoria: Republic of South Africa, 1995. Available at: https://www.saps.gov.za/legislation/acts/act68of1995.pdf accessed 09.04.2019).

### Problem definition: lack of household supply, alternative and unregulated sources

Repeated and prolonged periods without piped water were recorded as was widespread use of informal and unregulated sources, several of which were known to be contaminated: ‘*There is no water…we are using dirty water that we get in the rivers*’ (family member, workshop 2). Participants described regularly collecting water from neighbouring villages, boreholes or rivers, cement and traditional wells and surface sources (figure 2), which were distant from households and in which water was often contaminated or unavailable: ‘*I have a geyser [domestic electric water heater] in my house, because of salt water, it does not last…the electric kettle does the same. Salty water is not good*’ (community official, workshop 2). Domestic storage was also known to carry a high likelihood of contamination.

There were many reports of mobile water tankers delivering water to households provided by the municipality due to frequent interruptions in piped supply (figure 3). These were seen as a major problem. The mobile trucks were not always available due to lack of fuel, delivered contaminated water and corruption among drivers was common: ‘*… drivers of mobile trucks are segregating…they don’t give water to some people*’ (family member, workshop 2). Participants also reported that although tankers appear to relieve the problem, politicians take advantage of the situation by selling water to villages exorbitantly. There were concerns that water provision through tankers deters efforts to improve the situation and impedes provision of domestic taps.

### Causes and contributors of water shortages

Participants overwhelmingly related lack of water to poor planning by water authorities, municipality and service providers asserting that these groups are out of touch with realities on the ground: ‘*our Government … does not know what is happening in our communities…it is time for them to know about the water shortage problems*’ (family member, workshop 2). There was consensus that community leaders are unaccountable and lack authority and motivation. Local leaders were also reported to make election promises that were not honoured. ‘*We have been promised that we will get water … it’s been two years now and there is no water in those taps*’ (WRA, workshop 2). Corruption was a common concern: ‘*the leaders are not working for us…they are corrupt. If they see that what they are doing is wrong, they try to find ways of covering it up*’ (family member, workshop 2). The situation was reported to leave community members confused about the roles of community leaders and the authorities, and widespread lack of mutual understanding and trust was evident.

While many forms of infrastructure were described, boreholes, pumps and storages tanks, functionality was described as inconsistent and unreliable due to lack of maintenance. Taps were reported to run dry and break down, and reservoirs were reported to be empty due to malfunctioning pumps: ‘*They put the borehole here, but the pump is broken*’ (family member, workshop 3). Delays in maintenance, replacement and repairs were described, as was vandalism and limited community ownership related to frustration and disillusionment. Shortages were also reported to be exacerbated by high temperatures, persistent droughts and low rainfall resulting in depletion, salinisation and evaporation of dams and rivers: ‘*There is not enough rain, if it rains, we wouldn’t have serious issues*’ (WRA, workshop 2).

### Health and social impacts

There was consensus that the absence of clean water poses significant threats to well-being and survival. Avoidable infectious disease and mortality, waterborne diseases including schistosomiasis, cholera, typhoid and other intestinal infectious conditions were linked to lack of water. A range of contaminants in drinking water were reported including animal and human faecal matter, plastics and other pollutants. Sanitation and hygiene were reported to be lower when there is no water and that people are forced to compromise, sacrifice and recycle water. People were acutely aware of the health impacts of lack of a reliable clean water supply: ‘*we will die because of having no water. In previous years, we had water in the rivers but now there is no water…If there was water in the rivers, we could wash our clothes there but now we cannot, and we stay dirty*’ (family member, workshop 2).

Continuous water shortages were described as an overwhelming impediment driving people into poverty and hunger, as it becomes more difficult to prepare food without water, wash, do laundry, grow plants and work (figure 4): ‘*Poverty and hunger are also part of the effects… if we don’t have water…we cannot cook, bathe, clean our houses or do backyard vegetable gardens*’ (family member, workshop 5). Serious economic impacts were also acknowledged. Buying water from tanker drivers is unaffordable in areas of high unemployment and welfare dependence: ‘*our finances are also affected because we use our money to buy water… we should use that money to buy other things*’ (WRA, workshop 2). Life was described as an ongoing struggle without water especially for vulnerable community members: ‘*I am unable to push the wheelbarrow [disabled participant with one hand]. I am unable to carry the bucket of water on my head. I wish I could have a tap in my house…it will make my life easier*’ (family member, workshop 2).

The burden of collecting water was documented as primarily borne by women and children. Significant amounts of time were described walking long distances to collect and carry water. It was also reported that water is often only available for collection at night and early morning, which introduces safety risks (figure 5): ‘*at night it is difficult for us women to go there and collect water because we fear criminals. We need men or other people to accompany us*’ (WRA, workshop 2). There was agreement that time spent collecting water could be used for income generating and educational activities. Children were also noted to miss school and parents have less time to care for families due to the need to collect water: ‘*Education is affected… children won’t do their homework because they will have to go fetch water after school*’ (family member, workshop 5).

Water shortages were also considered as a significant source of personal unhappiness, stress and division in families and communities. Participants reported finding it embarrassing and disruptive to continually collect water, and conflicts and tensions were reported to arise between households over mobile water tankers, who gets water first and how much, with disputes about access with boreholes: ‘*they do provide us with mobile water tanks sometimes, but they are not helping except causing more fights and hatred among community members*’ (family member, workshop 4). Deficiencies in resource management and service delivery were also reported to result in frequent community protests, which often become violent.

Some aspects were conveyed differently between men and women. Several WRA participants described how women and girls have major roles in water collection, limiting participation in waged work and education and increasing risks of injury and violence. While there was relatively low male representation in the group in which water was nominated as the priority topic (approximately 20%), male participants spoke of causes, contributors and impacts related to poverty, poor infrastructure, unemployment, neighbourhood tensions and disconnection from the authorities. These perspectives were confirmed when all groups came together at the end of the process, although here the proportion of males was similar.

### Priorities for action

There was unanimity that taps in houses are urgently required as an overall goal: ‘*We do not want mobile water trucks but taps in each and every household*’ (WRA, workshop 4). To achieve this, actions were developed around improving reporting systems on the extent of the problem as a basis from which to advocate for fairer resource allocation. Participants identified improved planning to include: inventories of households without water; detailed monitoring and reporting of water-related challenges; fund-raising to support monitoring, planning and infrastructure development; and fairer allocation of resources through multisectoral deliberation and partnerships for infrastructure development and maintenance.

**Figure 6:**
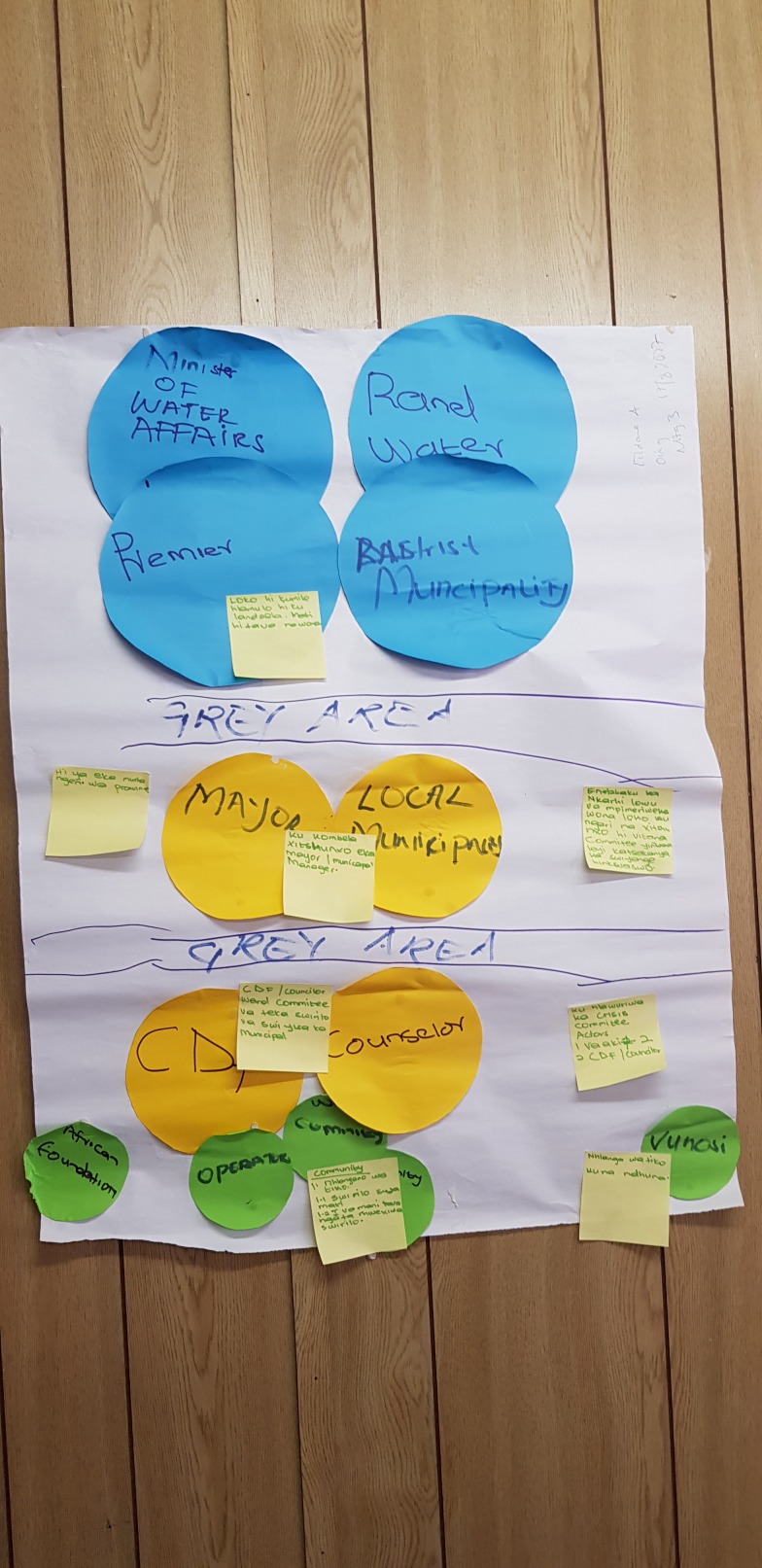
Venn diagram indicates lines of obscure communication (‘grey areas’) between different levels and sections responsible for water resource management and service delivery.

The lack of connection between communities and authorities was confirmed during this process, with lines of communication between different governing levels and sections described as ‘grey’ (figure 6). The importance of closer working links with local government and municipalities was therefore seen as necessary before engaging higher level stakeholders: ‘*we can’t start at the top ranks… those people can’t relate to the challenges that we face… we need to start with someone who knows our situation*’ (traditional healer, workshop 4). The need to improve knowledge of who to approach for broken pumps, leakages and illegal connections was also emphasised.

To this end, participants delineated the roles of existing community assemblies. Several community structures were described from which ward committees and community leaders (including village heads and Indunas with authority over several households in line with customary law) were identified as having important roles. Ward committees are able to raise priorities with the Community Development Forum (CDF), a group that addresses developmental issues in villages. The CDFs prioritise community needs by liaising with elected councillors, and the ward plan is then taken by the councillor to be amalgamated with priorities from other wards in the municipality to form an Integrated Development Plan: ‘*the Ward Committee is the structure that refers grievances to higher structures and authorities*’ (family member, workshop 4).

Strengthening infrastructure and maintenance and advancing technologies to bring an end to water distribution via mobile trucks were identified as critical. Participants also stressed that they need to be involved in all stages planning, implementation and management of water supply. Community awareness was identified as a complementary route to educate the community on the value of water, conservation and best practices such as waste disposal to avoid pollution and land degradation as well as the need to develop collective responsibility to protect catchment areas and existing infrastructure: ‘*we should be responsible when using* water’ (family member, workshop 2).

### Reflections on the process

**Figure 7:**
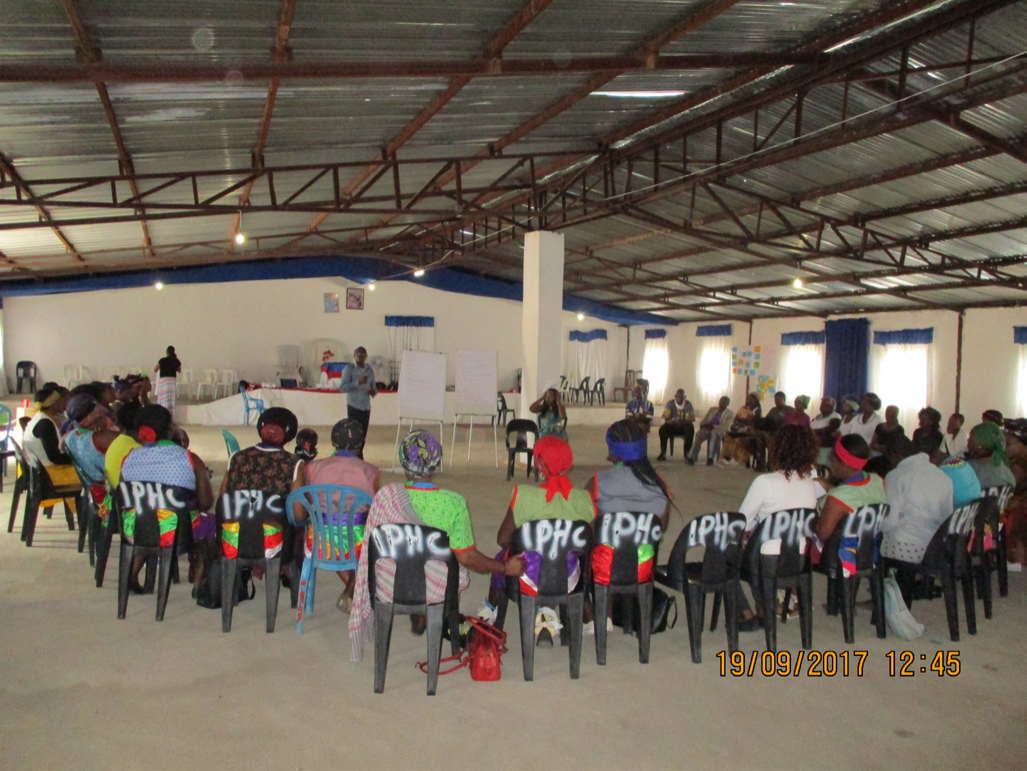
Participants from all villages convene to discuss and build consensus on nominated topics in session cofacilitated by community stakeholders.

**Figure 8:**
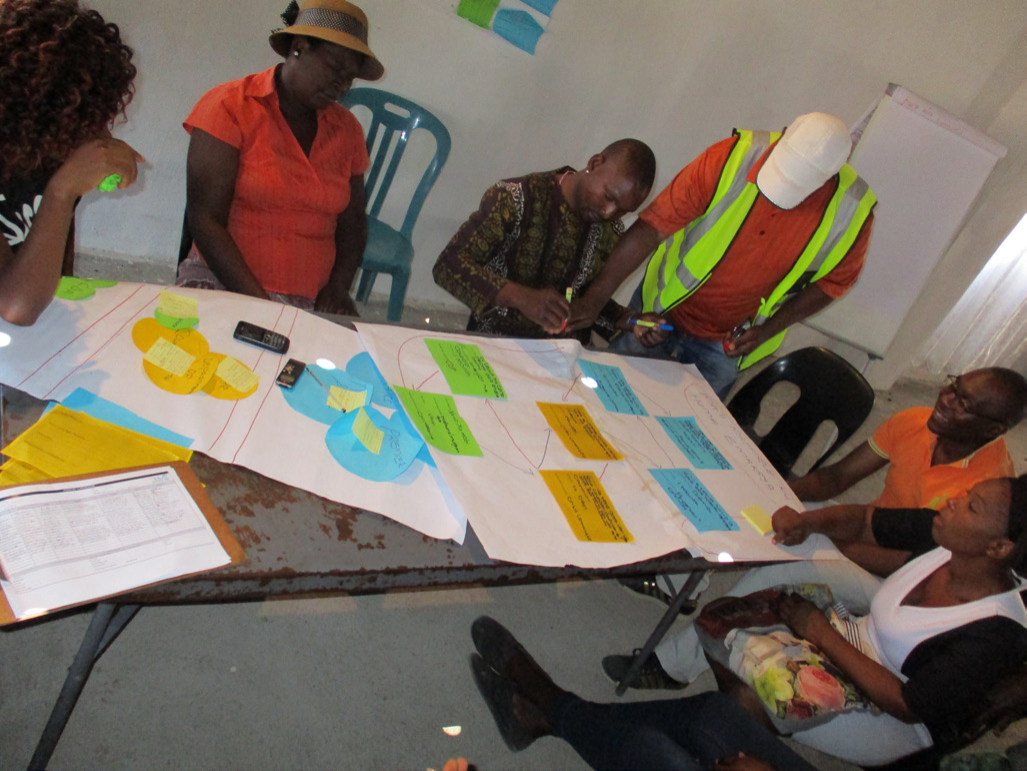
Development of action pathways based on workshop outputs detailing: problems, causes, impacts, actors and institutions.

The process created spaces for colearning, understanding others and facilitated new linkages. Participants were oriented to consensus-building techniques, which enhanced team work, took active roles during the weekly workshops, were keen to learn new skills and were generally enthusiastic about the approach (figures 7–8). Participants reported feeling valued and respected when they recognised that their contributions, voices and ideas were captured: ‘*we are happy because our views were considered*’ (family member, workshop 8). Attendance was high in all meetings; there were no drop outs other than one participant who relocated to Johannesburg.

Challenges were encountered in terms of expectations around action, with some participants anticipating improvements in water supply at the end of the eight weeks. There was also some dissatisfaction expressed round the level of reimbursement and indications that it should be higher. We regularly reviewed and rationalised expectations, resource availability and reflected on and revised the design, outputs and next steps while moving the work forward towards development of action plans with government and non-government actors: ‘*I think everything was good and we learnt a lot in our discussion. We will be happy if we achieve what we want to achieve. We will dance and ululate (loud sounds to express joy) for that like never before*’ (WRA, workshop 8).

## Discussion

In this section, we reflect on the findings, consider acceptability and reproducibility of the method, and outline learning and next steps engaging government and other agencies to incorporate community-generated evidence into resource management and service delivery.

### Substantive findings

Despite overall improvements in access reported in national data, our data suggest a very different picture. Everyday life was reported to be a continuous struggle without water, entrenching poverty, illness, hunger, stress and social unrest. While our participants were not a random sample, they were also not selected as a group with whom water shortages were pervasive. This suggests that aggregate statistics may not fully represent the reality of water access and associated challenges. The findings are consistent with other research on water security in rural South Africa, although on specific health and social impacts in isolation and that acknowledge the reality of water insecurity and benefits of interventions are likely to be underestimated.^46–48^ PAR yielded rich renditions of interconnected social, behavioural and health impacts, allowing fuller understandings of the true burden to be recorded.

PAR was adopted to understand and enable interfaces between communities and public authorities. As a first step, articulating needs and priorities among community stakeholders, there was unanimity that water provision can only be of benefit with taps in households. Recent research in the area has associated piped water provision with an eightfold reduction in child schistosomiasis, suggesting this recommendation may have transformative potential.^48^ Steps towards the goal were chosen to improve trust between communities and local planning, management, maintenance and surveillance. The plans were pragmatic and incremental, focused on existing community structures and generating evidence for planning, advocacy and resource allocation. Rural communities have effectively monitored water quality with new technologies elsewhere in South Africa suggesting there may be important roles for communities to support authorities in surveillance and monitoring.^20^ There was also willingness to develop awareness campaigns and education around water access and safety.

It was recognised that solutions cannot be achieved by organisations working in isolation. Multisectoral collaboration was seen as necessary to sustainably improve access through collective and coordinated efforts to understand demand, supply and equity issues and promote innovation in the face of climate change. International debates set out that multisectoral governance requires accountable institutions, clear roles and responsibilities and effective coordination. Challenges lie in complex mechanisms, coordination problems and conflicts of interest, resulting in failures to mediate differences between diverse actors.^49^ This is of critical importance in South Africa where community-based water governance has been significantly undermined by social and political power asymmetries accumulated over generations.^50^ As the process develops, there is a need to understand whether shifts in power relationships can occur, through which processes, in which contexts and with which outcomes.^19 51^ To this end, we reflect on the methodology and consider next steps below.

### Methodological reflections

According to Cook *et al*, PAR has an impact on the quality of research design when participants are actively involved in designing and directing the process.^52^ Participant involvement in key design elements (nomination of topics and expansion of participant base) embedded shared responsibility and ownership from the outset. Some participants were illiterate but participated actively in the process. While we adopted a predetermined sequence, the process embodied and reinforced principles of openness and respect for others, and skills to listen, observe, analyse and interpret throughout, which were well-received among community stakeholders.

The discussions were structured and sequenced to build collective understandings of key priorities faced in rural villages in a self-directed process. The problem tree was a simple, relatable framework that allowed an initial systemising of perspectives. From this, we progressed to more complicated tasks developing strategies for action. Venn diagramming was effective in encouraging participation to map relevant actors, institutions and inter-relationships. The action pathways were a demanding aspect given the extent of challenges identified. Drawing on the prior outputs of the problem tree and Venn diagram, however, it was possible to articulate shared goals and identify stepwise actions towards these.

Transformational learning happens when researchers and participants have sufficient time to interact. Participants and researchers engaged over a series of eight workshops. Repeated engagements supported development of rapport and relationships, as a foundation to progress through a sequence of increasing complexity. The approach also allowed principles of collaborative action and knowledge production to be examined and re-examined. Photovoice was accessible and stimulated active participation, participants confidently presented vivid visual evidence with pride and satisfaction. The final meetings were sufficiently familiar in process and principles for community stakeholders to adopt roles as cofacilitators (figure 7).

There were several challenges. There was some dissatisfaction with the level of reimbursement and expectations were raised around the likelihood of improvements in water security. We invested time rationalising expectations, were consistent and transparent in information on resources, next steps engaging with the authorities and the extent of community representation and ongoing communication in these. Despite these challenges, there was high and consistent engagement and generally positive feedback. While direct citizen action was not within the scope of the workshops, the process provided an acceptable means of capturing the multidimensional nature of the problem and developing community assemblies as a first step towards the provision of this information in the wider research programme.

### Next steps

The next steps are to engage decision makers to leverage action based on local needs. Overall, the programme aims to bring state and non-state actors together to address community-nominated priorities in a collaborative learning platform promoting knowledge transfer between scientific, policy and local communities. Diverse stakeholders and interests will inevitably introduce risks of reproducing social and political power asymmetries, and so sensitivity to power relations will be critical. As stated by Brown^50^ on the inherent and potentially limiting assumptions around participatory governance: ‘*creating new multi-stakeholder spaces is the easy* *part*’. Wider informal norms, trust and authenticity do not change rapidly, and there is a potential to recreate inequalities and power asymmetries.

Sensitivity to context and evaluation of the process, outcomes and limits of participation are therefore required as the process expands. In terms of process, attention to issues such as extent of codesign, loss of earnings, travel costs and suitability of venues, countervailing the power of dominant individuals and groups and to often bureaucratic and corrupt service and statutory contexts will be necessary. Measurement of outcomes to understand whether tangible changes are taking place, and whether and how they can be attributed to the process, will also be required to understand whether true shifts in power relationships and distribution of benefits are possible.^51 53^

The research environment is of critical importance in this regard. Neutral research spaces can provide a basis to reconcile diverse viewpoints, needs and goals. HDSSs offer important opportunities to build meaningful relationships and trust between communities and authorities, as well as provide timely and robust data for monitoring and evaluation. To our knowledge, this work is the first to develop deliberative decision making in water services through a demographic surveillance platform. Demographic surveillance system infrastructure is expanding in South Africa where there is recognition of their value in national planning and development.^54^ There may therefore be significant potential to improve interfaces and relationships between communities and authorities to mobilise and take up water monitoring activities through participatory research embedded in HDSS.

## Conclusion

Access to clean water requires effective governance under demanding conditions. Community participation can contribute evidence on the true scale of the problem and on how it can be resolved. While theoretically appealing, participation in water governance is neither widely conducted nor generally effective. HDSS research environments offer important opportunities to enable innovations in participatory governance, resolving gaps in planning and informing resource allocation. The outputs of the PAR process will form the basis of new engagements with government and NGOs engaging in adaptive learning processes to further explore community-led governance for water security. Sensitivity to multiple interests and actors, process and power dynamics, social, political institutional and cultural contexts, and outcomes will be necessary in the next stages.
